# Minority Identity, Othering-Based Stress, and Sexual Violence

**DOI:** 10.3390/ijerph19074221

**Published:** 2022-04-01

**Authors:** Lotte De Schrijver, Elizaveta Fomenko, Barbara Krahé, Kristien Roelens, Tom Vander Beken, Ines Keygnaert

**Affiliations:** 1 International Centre for Reproductive Health, Department of Public Health and Primary Care, Ghent University, 9000 Ghent, Belgium; elizaveta.fomenko@ugent.be (E.F.); ines.keygnaert@ugent.be (I.K.); 2 Department of Psychology, University of Potsdam, 14476 Potsdam, Germany; krahe@uni-potsdam.de; 3 Department of Obstetrics & Gynaecology, Ghent University Hospital, 9000 Ghent, Belgium; kristien.roelens@ugent.be; 4 Department of Human Structure and Repair, Ghent University, 9000 Ghent, Belgium; 5 Department of Criminology, Criminal Law and Social Law, Institute for International Research on Criminal Policy, Ghent University, 9000 Ghent, Belgium; tom.vanderbeken@ugent.be

**Keywords:** sexual orientation, poverty, minority health, sexual and gender-based violence, rape

## Abstract

Background: Some (minority) groups (MGs) are more vulnerable to sexual violence (SV) exposure than others. Othering-based stress (OBS) may mediate the relationship between minority identification and SV. This study aims to assess the prevalence of SV in different MGs to explore the relationship between minority identification and SV, to investigate whether belonging to multiple MGs moderates this relationship, and to explore OBS SV moderation for different MGs. Method: Through an online survey administered to a nationally representative sample in Belgium, data was collected from 4632 persons, of whom 21.01% self-identified as belonging to a MG (SI-Minority). SV prevalence was measured using behaviorally specific questions based on the WHO definition of SV. SI-Minority participants received an additional scale on OBS. Results: SI-Minority participants reported more SV victimization compared to the non-minorities. However, this increased risk was not moderated by minority identification but linked to the socio-demographic SV risk markers common to minority individuals. Multiple-minority participants were found more at risk of SV compared to single-minority respondents. Lesbian, gay, bisexual, pan-/omnisexual, asexual, and other non-heterosexual (LGB+) participants were found more at risk than heterosexual participants. OBS was found to be significantly correlated to SV in sexual and gender minorities and in cultural minorities. Conclusions: This study contributes to our understanding of the relationship between minority identification, OBS, and SV. Studying both specific and common SV vulnerabilities and outcomes within specific societal subgroups and the general population may inform policy makers when allocating resources to those interventions with the largest societal impact.

## 1. Introduction

Sexual violence (SV) includes “*every sexual act directed against a person’s will, by any person regardless of their relationship to the victim, in any setting*” [1] and consists of different hands-off types, such as sexual harassment without physical contact, and hands-on types, like sexual abuse with physical contact, but without penetration and (attempted) rape [2,3]. SV is a major public health issue that affects people worldwide, in every culture, and in every social layer of society [4,5]. Vulnerability to SV is influenced by factors occurring at an individual, interpersonal, community, and societal level [6,7]. These interacting factors increase or reduce the risk for SV exposure and can lead to (re)victimization, perpetration, or both [7]. The available evidence shows that people are more at risk of sexual victimization while they are younger, when they were assigned the female sex at birth and/or identify as a woman; when they were previously exposed to (in)direct violent experiences; when they have physical and/or mental health problems or are dependent on others for care; when they show risk behavior, such as harmful alcohol abuse, drug use, and risky sexual behavior; and when they report lower educational levels and socio-economic status [3,7,8,9,10,11,12,13,14,15,16,17,18,19,20,21,22,23,24,25,26,27,28,29,30,31,32]. On a community and societal level, gender inequality, ruling gender norms, ideologies about male sexual entitlement, rape myth acceptance, legal frameworks targeted at sanctioning sexual perpetration etc., are identified as common drivers for creating contexts in which SV occurs [7,33,34,35,36,37,38,39,40]. This list is not exhaustive, but mentions frequently observed risk markers in the general population.

### 1.1. Vulnerable Groups

Everyone is at risk of being exposed to some form of SV. Yet, some groups are more vulnerable to SV exposure than others. Migrants, applicants for international protection, and refugees (MARs), for instance, have been shown to be at increased risk [3,41,42,43,44,45,46,47], and the same is the case for sexual and gender minorities (SGM) [48,49,50,51,52,53,54,55,56,57,58,59,60,61].

When looking at the identified vulnerabilities for increased exposure to sexual victimization observed in MARs [41,46,62,63,64] and SGM [11,48,58,60,65,66,67,68,69,70,71,72,73,74,75,76,77,78,79,80,81], commonalities can be identified. In both populations, identifying with the female gender, having a lower socio-economic status, experiences with prior interpersonal violence, breaking traditional social roles expectations, and poorer (mental) health are identified as SV risk markers. Further, belonging to more than one minority group—including because of a migration background—was also identified as a risk marker in SGM [70,71,72].

As members of minority groups, MARs and SGM also have in common that they are susceptible to differential treatment in the societies in which they live [82,83] and are often confronted with stigmatization, prejudice, and discrimination [83,84,85,86,87,88]. Stigma, discrimination, and prejudice emerge when individuals and groups are ‘othered’. ‘Othering’ can be defined as a process that serves to mark and name those individuals who are considered to be different from oneself. It secures and defines a person’s or group’s identity through the stigmatization and distancing of others in an “us-them” separation [89]. The effect of the stress caused by being perceived as “different” on general stressors requires additional adaptation efforts to cope with stressful situations compared to non-stigmatized peers [83]. Although this type of stress is referred to as minority stress [83] in studies on SGM, in this study, we will use ‘othering-based stress’ instead. Othering-based stress (OBS) thus refers to the excess stress experienced by individuals who are exposed to othering practices, independent of their minority membership.

Recent evidence suggests a link between othering-based stress and an increased risk of experiencing SV in SGM. It also suggests that SV has a larger and more lasting impact and creates barriers for victims who seek help upon sexual victimization as well [61,69,90,91,92,93,94]. The combination of multiple minority identities may increase the impact and probability of sexual victimization [69]. Recognizing that minority individuals may be part of more than one minority group or may share some characteristics with multiple-minority groups and others with the dominant group is forwarded in the framework of intersectionality [95]. Hence, multiple-minority identities may yield different social experiences.

Most SV research has focused on one minority group at a time. Yet, the identified vulnerabilities might be common to other minority groups as well. A lower socio-economic status and economic poverty, for example, have also been observed in minorities such as people with a disability [96], especially when multiple minority identities intersect [97]. Earlier studies have shown a clear link between socio-economic status and poverty with SV. SV can jeopardize a person’s economic wellbeing, potentially leading to homelessness [98,99], unemployment, interrupted education and health, mental health [8,64,98,100,101], and other daily stressors and struggles which impact one’s socio-economic status. In turn, not being able to meet one’s basic needs can increase a person’s vulnerability to sexual victimization [101,102]. Those individuals who appear vulnerable—whether because of their gender, age, skin color, disability, sexual orientation, gender identity, migration background, income, or other reason—are exploited and victimized, and survivors get caught in vicious cycles created by poverty and other negative consequences of SV and othering [47,62,101]. People who belong to a minority group—independent of the minority characteristic—might thus be vulnerable to sexual victimization. Yet, to date, the underlying mechanisms to the link between sexual victimization and belonging to a minority group remain unclear.

This study wants to go beyond studying individual minority groups by looking into this common vulnerability of minorities in Belgium. We considered it key to explore whether the observed vulnerabilities are specific to these populations or whether they may be common to several minorities—including MARs and SGM, but not limited to those two groups—in general. We want to explore whether other minorities who may also be exposed to othering practices experience stress linked to stigma, prejudice, and discrimination, and we want to know whether there is a relationship between experiencing othering-based stress and SV in different minority groups.

### 1.2. Current Study

With this study, we aim to extend the research base regarding SV in minority groups. The research objectives are to analyze the prevalence of SV in different types of minority groups in Belgium (1) and the difference between people who identify with a minority group and those who do not (2). We hypothesize that minority participants will report more SV than non-minority participants. Further, we want to investigate whether belonging to multiple minority groups moderates the relationship between SV and minority identity (3). Hereto, we compare participants who indicated to belong to a single minority group with those who indicated to be part of two or three different minority groups. We hypothesize that the latter group is more at risk of SV compared to single-minority participants. Individuals who experience OBS may become more at risk of sexual violence because OBS impacts coping and health-seeking behavior both via dynamics of interpersonal discrimination and via internalized oppression and stigma. Maladaptive coping strategies (e.g., alcohol and drug abuse; hypersexual behavior; etc.) as well as not seeking help upon victimization, in turn, increase the vulnerability to sexual victimization. Lastly, we want to explore whether OBS is associated with sexual victimization for the different studied minority groups (4). The hypothesis here is that experiencing OBS will be significantly linked to an increased SV exposure.

## 2. Methods

### 2.1. Sampling Procedure and Participants

As part of a broader mixed-methods research project called ‘UNderstanding the MEchanisms, NAture, MAgnitude and Impact of Sexual violence in Belgium’ (UN-MENAMAIS) [103], data were collected via a cross-sectional quantitative study using an online survey administered to a nationally representative sample of Belgian citizens aged 16 up to 69 years old. The Belgian National Register (BNR), containing demographic information on all Belgian residents, served as the sampling frame for two independent waves of data collection in the national representative sample. A random disproportionate stratified sample was drawn from the BNR with the aim to reach an equal number of male and female participants equally divided into three age groups (i.e., 16–24 years old, 25–49 years old, and 50–69 years old). This sampling method allowed us to overrepresent certain subgroups in the first data collection wave to guarantee sufficient statistical power for each relevant subgroup. This overrepresentation was corrected post-hoc in the second wave, using survey weights to obtain estimates representative of the population residing in Belgium (see [8] for more details).

The required sample size was calculated with a 2% margin of error and a significance level of 5%. Based on previous research on SV prevalence in Belgium (e.g., [34]), the expected prevalence rate was estimated at 10%. The sample size calculations led to an a-priori total sample size of 5190 participants with a targeted 864 participants per subgroup. To reach this target while considering potential non-response and refusals to participate, four times the estimated required sample size was invited for participation (i.e., *n* = 20,760 for each of the the two data waves).

The main goal of the quantitative study was to conduct a pre-validated self-report survey probing into sexual victimization and perpetration among a randomly selected sample of Belgian residents aged 16 to 69, regardless of their gender or sexual orientation (see [3]). To limit self-selection bias, the study was presented as a broader survey about health, sexuality, and wellbeing. The online survey was administered via the survey software Qualtrics (Qualtrics, Provo, UT, USA). Participants could access the self-administered survey using either a link or a Quick Response (QR) code that could be scanned using a smartphone, indicated in the letter sent by the BNR. Before participation, potential participants received additional information on the study and an informed-consent form. Only upon informed consent were respondents able to proceed in the survey. To increase response rates, sampled potential participants received one reminder letter two weeks after their initial invitation and were informed about the possibility of receiving a randomly assigned voucher worth 30 EUR upon participation. For partake in the latter, participants were directed to a separate short questionnaire after completing the main survey to ensure that survey answers could not be linked to personal contact information.

Within the period between 10 October 2019 and 1 January 2021, two independent waves of data collection took place. A total of 41,520 Belgian residents between 16 and 69 years were contacted by the BNR by post.

### 2.2. Measures

#### 2.2.1. Assessment of Sexual Violence

In this study, SV was defined according to the WHO definition (cf. supra) [1]. As is recommended internationally [104,105,106,107], behaviorally specific questions were used to provide reliable estimates of both female and male sexual victimization [107] for participants of different sexual orientations or gender identities or different cultures. The details of the validation procedure are described elsewhere (see [107,108]).

The questionnaire was designed to maximize SV (victimization and perpetration) disclosure by starting with less sensitive topics building up towards the questions regarding SV. Lifetime SV experiences were assessed. The behaviorally specific questions were derived from the revised Sexual Experience Survey (SES-R) [105,109], the Sexual Aggression and Victimization Scale (SAV-S) [110], and the Senperforto questionnaire [44]. All questions on SV were adapted to the Belgian social and legal context. The process of developing this survey is described elsewhere (see [3,107]). Only the victimization reports were included in the current analysis.

#### 2.2.2. Assessment of Minority Identity

Every participant was asked to indicate whether they considered themselves to belong to a minority group in Belgium and, if so, to indicate to which characteristic (i.e., sexual orientation, gender identity, intersex or differences of sexual development (DSD) condition, religion, or life philosophy, skin color, ethnicity, disability, age or another characteristic) this is related. Multiple answers were possible. Based on the answers, we created four comparison groups for the analysis: (1) ‘sexual and gender minorities’ (SGM), i.e., all individuals who selected at least one of the following characteristics: sexual orientation, gender identity, and/or being an intersex/DSD person; (2) ‘cultural minorities’, which grouped all participants who identified with a minority group because of their ethnicity, skin color and/or religion/life philosophy; (3) ‘other minorities’ for all participants who indicated that they belonged to a minority group because of a disability, their age, and/or another characteristic; and lastly (4) ‘multiple minority’, which grouped those participants who indicated to belong to a minority group related to at least two of the three earlier mentioned groups.

#### 2.2.3. Assessment of Othering-Based Stress

Participants who indicated to belong to a minority group in Belgium because of their sexual orientation, gender identity, intersex or DSD condition, religion or life philosophy, skin color, and/or ethnicity received the Othering-Based Stress Scale (OBS-S)—an adapted version of the minority stress measure validated in the United States [111]—relevant to the characteristic they had indicated. Participants who did not identify as a member of a minority did not receive this scale.

The OBS-S consisted of six subscales with a total of 25 5-point-Likert-items (ranging from 1 = Strongly disagree to 5 = Strongly agree): identity concealment (3 items), micro-aggressions (3 items), rejection anticipation (3 items), victimization events (10 items), internalized stigma (3 items), and community connectedness (3 items). The items from the last subscale, community connectedness, were rescaled (5 = Strongly disagree to 1 = Strongly agree) before creating a mean across all items (Cronbach’s alpha = 0.794) where higher scores indicate greater othering-based stress. Total scores greater than 4 indicate a high level of othering based stress. The OBS-S was used to assess minority stress experienced in relation to either ‘SOGI-related’ characteristics (i.e., sexual orientation, gender identity, and/or intersex or DSD condition) or ‘cultural-related’ characteristics (i.e., religion or life philosophy, skin color, and/or ethnicity). This newly developed version of the scale was validated for face-validity in the Belgian context (see [107,108] for more details).

### 2.3. Ethical Considerations

This study was approved by the Commission for Medical Ethics of Ghent University Hospital/Ghent University (B670201837542). It was designed and performed in line with the principles of the Declaration of Helsinki. This study only included participants of 16 years and older given ethical and practical regulations related to the legal age of consenting to sex, which is 16 years in Belgium. All participants gave informed consent before initiating the online survey.

## 3. Analysis

All analyses were run in R4.1.1. Descriptive statistics (means, standard deviations, counts, and percentages) were computed for all variables figuring across all tables. Significant differences in the distribution of nominal variables between (1) SI-Minority and non-SI-minority, and between (2) the four minority groups (sexual and gender, cultural, other, and multiple) were computed using (post-hoc) chi-square-tests. If the assumptions were not met, a Fisher’s Exact Test was used. To compare the mean age, the independent samples t-test was used. All assumptions were checked. Levene’s Test was used to check for homogeneity of variance, which led to the use of the Welch’s *t*-test statistic, as equal variances could not be assumed. Two binary logistic regressions were calculated to analyze the association between socio-demographic variables (such as in Table 1), self-identification with a minority group, and the prevalence of lifetime hands-off and hands-on SV. To avoid multicollinearity, the correlations were checked between all variables. To ascertain the power required for the multiple logistic regression analyses, we assumed a ratio of 10 cases per predictor, based on the simulation by Peduzzi et al. [112]. This condition was satisfied for all analyses. There were no strong correlations between the predictors. Self-identification with a minority group (no, sexual and gender minority, cultural, other, and multiple minorities) was added as a moderator in the relation between the socio-demographic variables and the two outcome variables (hands-off and hands-on SV). Interaction terms with *p* < 0.05 were included in the model. A third binary logistic regression was used to analyze the association between OBS-S and the prevalence of lifetime SV. Finally, the odds ratio was calculated with its 95% confidence interval (CI).

## 4. Results

### 4.1. Sample

The online survey was started by 6504 respondents. Respondents were excluded because they did not give informed consent (*n* = 706), did not fully complete the survey (*n* = 909), did not meet criteria regarding age (i.e., between 16 and 69 years old; *n* = 6), completed the survey multiple times (*n* = 37), or because there were concerns about the quality of the responses (*n* = 1). Respondents who had missing values on key variables for this study (e.g., items on sexual orientation) were excluded as well (*n* = 213). The total final sample consisted of *n* = 4632, which corresponds to a response rate of 11.16%. We included 2018 participants from the first wave and 2614 participants from the second wave of data collection.

The total sample consisted of 2300 persons who were assigned the male sex at birth and 2332 persons who were assigned the female sex at birth.

The mean age of the sample was 39.07 years (SD = 17.02). In this sample, 4108 participants were born in Belgium. Of those who were not born in Belgium, 231 persons held Belgian nationality at the time of the survey. Further, 1020 persons had at least one parent who was not born in Belgium, and 1316 persons had at least one grandparent who was not born in Belgium. The survey was completed 2886 times in Dutch, 1578 times in French, 154 times in English, nine times in Arabic, and five times in Farsi. No one completed it in Pashtu.

Table 1 summarizes the socio-demographic characteristics of the unweighted sample. The self-identified minority group differed significantly from the group of participants who did not indicate to belong to a minority group in Belgium in terms of socio-demographic characteristics.

In comparison to publicly available information on the level of education in the entire population, people with higher levels of education were overrepresented in your sample. Almost half of all respondents (i.e., 49.89%) completed a level of higher education, while—on the population level—37.6% of Belgian residents between 15 and 64 years completed a higher educational level [113]. The comparison of the distribution of men and women across different age groups in the entire population aged 16 to 69 using publicly available data and those in our sample is presented in Table 2.

### 4.2. Minorities

In total, 21.01% of the participants (*n* = 974) identified as belonging to a minority group in Belgium because of their sexual orientation, gender identity, intersex or DSD condition, religion or life philosophy, skin color, ethnicity, disability, age, or another characteristic. Table 1 presents the socio-demographic information of this subsample.

An overview of the proportion of participants in this nationally representative study who identified as belonging to a minority group because of one or more specific characteristics that distinguish them from the majority of people living in Belgium is presented in Table 3. About one in five participants indicated to self-identify with at least one minority group. Most of them identified with a cultural minority (10.31%) because of their ethnicity (5.29%), skin color (4.19%), and/or religion/life philosophy (5.72%). We will further refer to this group as ‘cultural minorities’. In this sample, 3.30% said that they belonged to more than one minority group (i.e., multiple minority identities): 34 participants indicated to be part of a sexual and gender minority as well as a cultural minority; 32 participants indicated both a sexual and gender minority and an ‘other’ minority; 68 persons combined a cultural minority identity with an ‘other’ minority, and finally 19 persons identified with all three minority groups. Most multiple-minority participants indicated thus to possess an ‘other minority’ characteristic.

The second-largest minority group (8.72%) consisted of persons who indicated their disability (2.42%), age (2.89%), or another characteristic (3.97%) to distinguish them from the Belgian majority.

Sexual and gender minorities (SGM) (5.61%) were the smallest group in this sample, with 5.48% of respondents stating their sexual orientation, 0.56% their gender identity, and 0.02% being intersex or having a difference of sex development as the distinguishing factor.

### 4.3. Prevalence of Sexual Victimization

In Table 4, we present an overview of the reported lifetime prevalence of both hands-off and hands-on sexual victimization in the total sample, the subgroup who identified as belonging to a minority group, and those participants who did not identify with a minority group. With the exception of exposure to unwanted sexual images, being forced to kiss someone, exposure to unwanted touching during care, and being unwantedly penetrated, self-identified minorities reported significantly more exposure to any type of SV compared to the non-minority. Identifying with a minority group seems to be a moderator of the probability of sexual victimization.

Table 5 shows the distribution for any sexual violence, hands-off sexual violence, hands-on sexual violence, sexual abuse, and rape for lifetime occurrence per minority group. Minority groups differed significantly in terms of lifetime SV prevalence. The results of the post-hoc χ^2^ tests showed that the SGM and the multiple-minority groups had higher overall rates of sexual victimization compared to the cultural and other minority groups, but did not differ amongst each other. SGM participants experienced significantly more hands-off SV than cultural and other minority participants. The multiple-minority group experienced more hands-off SV than the cultural-minority group. The multiple minority groups were found to be more exposed to hands-on SV compared to the cultural and another minority group. The reported prevalence rates for any type of SV for cultural and other minorities are similar to those of the non-SI-Minority group (see Table 4; rate of 62.38%).

Table 6 shows the results of the two logistic regression analyses. All socio-demographic variables were found to be significantly associated with hands-off and hands-on SV, except for educational level and occupational status that were only found to be significantly associated with hands-off SV. Respondents who were assigned the female sex at birth, were younger, perceived their financial situation as difficult, self-identified as being LGB+, or that self-identified as being part of multiple minority groups had a higher probability of both hands-off and hands-on SV. Respondents who were actively working or selected at least one of the characteristics of the other minority group had a higher probability of experiencing hands-off SV. SI-LGB+ respondents had 1.68 times higher risk of hands-off and 1.85 times higher risk of hands-on sexual victimization than SI-heterosexuals, if all other charateristics were kept the same. Respondents in the multiple-minority group had higher risk of hands-off and hands-on SV than respondents who did not self-identify with a minority group, with the odds being respectively 1.64 and 1.77. None of the interaction effects were found to be significant (*p* > 0.05), which means that self-identifying as being part of a minority group did not moderate the relation between the socio-demographic variables and SV. The association between sexual orientation and SV was therefore not different between respondents who self-identified as being part of the sexual and gender minority group and those who did not.

### 4.4. Othering-Based Stress and Sexual Violence

Respondents who self-identified as belonging to the SGM group reported a mean of 1.88 (SD = 0.41) on the OBS-S (with scores ranging from 1 to 5 and higher scores indicating greater othering-based stress). None of the respondents scored higher than 3.20, which means that no one reported a high level of othering-based stress (OBS-S value > 4) in this group. Respondents who self-identified as belonging to the cultural minority group reported an average of 1.84 (SD = 0.52) on the OBS-S, with only one respondent reporting a high level of othering-based stress.

A binary logistic regression model showed othering-based stress (Estimate = 1.264; Standard Error = 0.457; *p* = 0.006) to be significantly associated with lifetime SV in the SGM group. The odds of victimization increased significantly as the value on the OBS-S scale went up. The odds of sexual victimization increased by 3.54 (95% C.I. 1.49–9.01) per one extra score on the OBS-S scale. SGM respondents with, for example, a score of 2 thus had approximately a 3.54 times higher probability of falling into the victim group compared with respondents with a score of 1. Othering-based stress was also a significant predictor of sexual victimization in the cultural minority group (Estimate = 0.641; Standard Error = 0.230; *p* = 0.005). The odds of sexual victimization increased by the factor 1.90 (95% C.I. 1.23–3.03) per 1 unit increase in OBS. A comparison of the 95% confidence intervals of the odds ratios for the SGM and the cultural minority groups showed that the effect of OBS on the prevalence of lifetime SV is not significantly different between these two groups.

## 5. Discussion

In this study, we explored whether belonging to one or more minority group(s) in Belgium increased the probability of sexual victimization (cf. research questions 1, 2, and 3). Hereto, we analyzed the prevalence of sexual victimization in different minority groups in Belgium and compared it to the prevalence in non-minority participants. Our results showed that people who self-identify as belonging to a minority group are significantly more likely to report sexual victimization than participants not seeing themselves as part of a minority. However, binary logistic regression analyses showed that these increased odds are not predicted by the minority identification but rather linked to the socio-demographic characteristics minority individuals have in common, which serve as SV risk markers. The higher SV prevalence observed in the self-identified minority group compared to the SV prevalence in the group of participants who do not identify with a minority group appears to be due to the significantly different composition of these subgroups of our nationally representative sample. The strongest predicators of sexual victimization identified in this study were: perceiving one’s financial situation as difficult, self-identifying as LGB+, and belonging to multiple minority groups. Further, in line with previous studies in the general population, we found being female (as assigned at birth) and being younger to increase the odds of sexual victimization (see among others: [7,8,10,101]). This study confirms that persons who belong to a minority group possess more characteristics that make them vulnerable to sexual victimization.

When we look at the LGB+ participants in our sample, they appear more likely to experience SV regardless of their identification as minority or not. Persons who identified as sexual and gender minority seemed to be more at risk of SV exposure according to the univariate analyses, but this observation is likely the result of the high number of LGB+ identifying in this subgroup. LGB+ identification seems to be a predictor of both hands-off and hands-on SV. Furthermore, identifying with a minority group because of religion or life philosophy, skin color, or ethnicity did not yield more reported exposure to SV upon the binary logistic regression analysis. Yet, when participants indicated to belong to a minority group because of a disability, age, or another characteristic or to belong to a total of at least two minority groups, they had a higher risk of SV.

Our fourth research question entailed looking into the hypothesis that othering-based stress would be associated with an increased vulnerability of minority groups to sexual violence. In contrast with what we expected based on the literature, sexual and gender minorities and cultural minorities in our sample did not report high levels of othering-based stress, as defined by a mean score > 4 on the five-point scale. Nevertheless, as we hypothesized in line with other studies [90,91,92,93,94], othering-based stress is positively associated with sexual victimization observed in minority groups. The finding that OBS predicted the odds of sexual victimization despite the moderate overall level of OBS in the SI-minority sample suggests that experiencing othering status may be a powerful vulnerability factor. Yet, based on our data, we cannot confirm causality. Our data also shows that othering-based stress does not have a different effect on the prevalence of sexual violence for sexual and gender minorities and cultural minorities.

### 5.1. Limitations

Our study had some limitations that should be discussed. First, the OBS-S scale used to measure othering-based stress was only validated on face-validity in the Belgian population. Thorough testing of the validity and reliability of this scale is needed and recommended in other contexts as well.

Second, in this study, othering-based stress was only assessed in those participants who indicated to belong to a cultural and/or sexual and gender minority group. Since othering-based stress was found to be associated with sexual victimization and since we found among others that not all LGB+ persons identified with a minority group because of their sexual orientation, we cannot make statements about the role of stigma, prejudice and discrimination, and associated othering-based stress in LGB+ individuals’ increased risk of experiencing SV. As such, studying the level of othering-based stress in all subgroups of nationally representative samples could allow us to draw more comparative conclusions and to better understand who experiences othering-based stress and could therefore be more at risk of experiencing sexual violence.

Third, the response rate of 11.16% is lower than the 25% we anticipated based on earlier studies on sensitive issues such as sexuality and violence. Participation in survey research has generally declined in the past decades [114,115,116,117], and our survey topic was quite sensitive, which may have lowered the invitees’ willingness to participate. Although their anonymity was guaranteed, since potential participants were invited through the Belgian National Register at their home address, it may not have felt anonymous for everyone. Further, by inviting participants via regular mail to participate in an online survey, the extra step introduced to access the survey may have created an additional participation barrier. Because of the changes in the EU law on data protection [118], sampling information drawn from the Belgian National Register could not be shared with the researchers, limiting the number of reminders that could be sent to the potential participants and making it impossible to conduct a non-response analysis.

A low response rate is often considered as resulting in low-quality biased data [114]; however, based on the distribution of participants over the different socio-demographic categories and the comparison made on the basis of nationally representative data, we believe that response rate bias is limited in this study, and the quality of the data remains sufficiently high. Nevertheless, being careful when interpreting this data as nationally representative remains important. In addition, although we used a nationally representative sample that considered equal spread over different age categories and was equally balanced over the female and male sex at birth, the sample showed an overrepresentation of Dutch-speaking participants. This may have led to distorted results due to cultural differences that may be present in the different Belgian regions. Lastly, the general population sample balanced by age and gender included a minority subsample which was less balanced. For instance, the minority groups contained more participants who were given the male sex at birth and a larger proportion of participants from younger age categories. Although the binary logistic regression analyses took these differences into account, the latter may have influenced the findings regarding sexual violence prevalence since a higher occurrence of sexual violence was observed in the younger age categories [8], and recall bias may have influenced the reported incidents. Future studies could benefit from using matched samples in comparing the minority groups with their non-minority peers.

### 5.2. Research, Prevention, and Policy Implications

Based on our observations in this nationally representative sample, we encourage researchers to study not only risk markers and outcomes of sexual victimization within specific subgroups of societies but also to study commonalities observed in multiple groups. When designing, developing, and implementing primary, secondary, and tertiary sexual violence prevention strategies, budgetary and resource constraints always need to be considered. By focusing on both common and specific vulnerabilities in research, we help to inform policy makers in allocating resources to those interventions with the largest impact on a societal level. Moreover, by differentiating between common and specific vulnerabilities, prevention strategies targeting specific vulnerabilities can be tailored to cover what universal interventions may miss.

Further research is needed to help improve our understanding of the role of othering-based stress in the vulnerability to sexual victimization and to inform the development of prevention and intervention programs addressing othering-based stress and related vulnerabilities. The model of intersectional stress and trauma proposed by Ching et al. (2018) may serve as a theoretical base to further explore this relationship. Moreover, to be fully inclusive, further research is needed to determine what the characteristics may be that result in Belgian residents experiencing othering-based stress. In addition, more research is needed to understand the underlying mechanisms at play in the SV vulnerability of persons who perceive their financial situation as difficult. Earlier research already suggested that people with less financial means, tenants, people depending on others for housing, and homeless people may be particularly at risk [63,101,102,119].

## 6. Conclusions

This study contributes to the understanding of the relationship between minority identification, othering-based stress, and SV. Persons who identify as belonging to a minority group report more sexual victimization compared to persons who do not identify as such. Yet, the observed increased risk was not found to be linked to the minority identification, but rather to the characteristics minority persons often have in common and that make them vulnerable to sexual victimization. In addition, identification as LGB+, identifying with multiple minority groups or with a minority group because of a disability, one’s age or other characteristics not mentioned in our list served as specific risk markers for sexual victimization. Further, othering-based stress is positively associated with sexual victimization observed in minority groups. Studying both specific and common SV vulnerabilities and outcomes within specific societal subgroups and the general population may thus inform policy makers when allocating resources to those interventions with the largest societal impact.

## Figures and Tables

**Table 1:** Sample composition (*n* = 4632; unweighted). Socio-demographic information presented for the total sample and for persons who self-identified as being part of a minority group (SI-Minority) and those who did not self-identify as belonging to a minority group (non-SI-Minority).

Variable	Total NR Sample (*n* = 4632; 100%) *n* (Valid %)	SI-Minority (*n* = 974; 21.01%) *n* (Valid %)	Non-SI-Minority (*n* = 3658; 78.99%) *n* (Valid %)	X^2^; df; *p*-Value
**Sex assigned at birth**				
Female	2332 (50.35)	437 (44.87)	1895 (51.80)	14.81; 1; <0.001
Male	2300 (49.65)	537 (55.13)	1763 (48.20)	
**Age groups** [mean(SD)]	39.07 (17.02)	31.90 (15.58)	41.00 (16.88)	15.92; 1634; <0.001 *
16–24 years old	1452 (31.35)	486 (49.90)	966 (26.41)	
25–49 years old	1548 (33.42)	313 (32.13)	1235 (33.76)	
50–69 years old	1632 (35.23)	175 (17.97)	1457 (39.83)	
**Educational level**				
Primary education or none	281 (6.07)	93 (9.55)	188 (5.14)	54.20; 2; <0.001
Secondary education	2040 (44.04)	484 (49.69)	1556 (42.54)	
Higher education	2311 (49.89)	397 (40.76)	1914 (52.32)	
**Occupational status**				
Remunerated workforce	2347 (50.67)	344 (35.32)	2003 (54.76)	197.54; 2; <0.001
Student	1198 (25.86)	418 (42.92)	780 (21.32)	
Other	1087 (23.47)	212 (21.76)	875 (23.92)	
**Financial situation**				
Perceived as difficult	3401 (73.42)	629 (64.58)	2772 (75.78)	49.45; 1; <0.001
Perceived as easy	1231 (26.58)	345 (35.42)	886 (24.22)	
**Gender**				
Man	2282 (49.27)	529 (54.31)	1753 (47.92)	<0.001 °
Woman	2316 (50.00)	421 (43.22)	1895 (51.80)	
Transman	5 (0.11)	4 (0.41)	1 (0.03)	
Transwoman	1 (0.02)	1 (0.10)	0	
Other	28 (0.60)	19 (1.95)	9 (0.25)	
**Sexual orientation**				
Self-identified heterosexual	4168 (89.98)	663 (68.07)	3505 (95.82)	657.02; 1; <0.001
Self-identified LGB+ ^1^	464 (10.02)	311 (31.93)	153 (4.18)	

* Independent sample t-test with equal variances not assumed (instead of χ^2^ test): t; df; *p*-value. ° Fisher’s Exact Test (instead of χ^2^ test): *p*-value. ^1^ Participants who labeled themselves as: lesbian, gay, bisexual, omni-/pansexual, asexual, or other. Notes: Because the comparisons in this table involved 7 independent tests, we adopted a Bonferroni-corrected significance level of 0.05/7 = 0.007 for these analyses. Abbreviations: NR: Nationally Representative; SI: Self-Identified; SD: Standard Deviation; LGB+: lesbian, gay, bisexual, pan-/omnisexual, asexual, other; df: degrees of freedom.

**Table 2:** Sample weights. A comparison of the gender distribution between the Belgian population and the study sample stratified by age.

Age Group	Sex at Birth	Population *n*	Population Proportion	Sample *n*	Sample Proportion	Population/Sample = Weights
16–24 years old	Female	576,098	0.07	699	0.15	0.47
	Male	601,426	0.08	753	0.16	0.5
25–49 years old	Female	1,864,081	0.24	815	0.18	1.33
	Male	1,883,527	0.24	733	0.15	1.6
50–69 years old	Female	1,475,820	0.19	818	0.18	1.05
	Male	1,458,421	0.19	814	0.18	1.05
Total		7,859,373	1	4,632	1	

**Table 3:** Proportion of participants self-identifying as belonging to a minority group in Belgium.

	Total NR Sample (*n* = 4632) *n* (Valid %)
**Self-identified as belonging to a minority group in Belgium**	
Yes	974 (21.01)
No	3658 (78.99)
**Relevant characteristic for identifying with a minority group in Belgium**	
My ethnicity	245 (5.29)
My skin color	194 (4.19)
My religion/life philosophy	265 (5.72)
My sexual orientation	254 (5.48)
My gender identity	26 (0.56)
Being an intersex/DSD person	1 (0.02)
A disability	112 (2.42)
My age	138 (2.98)
Other characteristic	184 (3.97)
**Total sexual and gender minority ^1^**	260 (5.61)
**Total cultural minority ^2^**	478 (10.31)
**Total other minority ^3^**	404 (8.72)
**Total multiple minority ^4^**	153 (3.30)

^1^ Individuals who selected at least one of the following characteristics: Sexual orientation, gender identity, and/or being an intersex/DSD person. ^2^ Individuals who selected at least one of the following characteristics: Ethnicity, skin color, and/or religion/life philosophy. ^3^ Individuals who selected at least one of the following characteristics: Disability, age, and/or other characteristics. ^4^ Individuals who are part of at least two of the above mentioned total groups: Total Sexual and gender minority, total cultural minority, and/or total other minority. Abbreviations: DSD: Disorder of Sex Development; NR: Nationally Representative.

**Table 4:** Lifetime sexual victimization.

Item	Total NR Sample (*n* = 4632; 100%) *n* (Valid %)	SI-Minority (*n* = 974; 21.01%) *n* (Valid %)	Non-SI-Minority (*n* = 3658; 78.99%) *n* (Valid %)	X^2^; df; *p*-Value
**Any SV**	2965 (64.01)	683 (70.12)	2282 (62.38)	20.00; 1; <0.001
**Any Hands-Off SV**	2729 (58.92)	652 (66.94)	2077 (56.78)	32.81; 1; <0.001
Sexual staring	1832 (39.55)	444 (45.59)	1388 (37.94)	18.78; 1; <0.001
Sexual innuendo	1615 (34.87)	381 (39.12)	1234 (33.73)	9.81; 1; 0.002
Showing sexual images	835 (18.03)	266 (27.31)	569 (15.55)	71.93; 1; <0.001
Sexual calls or texts	575 (12.41)	180 (18.48)	395 (10.80)	41.75; 1; <0.001
Voyeurism	133 (2.87)	45 (4.62)	88 (2.41)	13,52; 1; <0.001
Distributing sexual images	79 (1.71)	21 (2.16)	58 (1.59)	1.49; 1; 0.222
Exhibitionism	652 (14.08)	192 (19.71)	460 (12.58)	32.40; 1; <0.001
Forcing to show intimate body parts	265 (5.72)	89 (9.14)	176 (4.81)	26.69; 1; <0.001
**Any Hands-On SV**	1445 (31.20)	357 (36.65)	1088 (29.74)	17.11; 1; <0.001
**Any Sexual Abuse**	1322 (28.54)	320 (32.85)	1002 (27.39)	11.25; 1; <0.001
Kissing	747 (16.13)	183 (18.79)	56 (15.42)	6.46; 1; 0.011
Touching in care	354 (7.64)	92 (9.45)	262 (7.16)	5.68; 1; 0.017
Fondling/rubbing	740 (15.98)	194 (19.92)	546 (14.93)	14.28; 1; <0.001
Forced undressing	207 (4.47)	62 (6.37)	145 (3.96)	10.39; 1; 0.001
**Any Rape**	509 (10.99)	154 (15.81)	355 (9.70)	29.32; 1; <0.001
Oral penetration	195 (4.21)	54 (5.54)	141 (3.85)	5.44; 1; 0.020
Attempt of oral penetration	190 (4.10)	75 (7.70)	115 (3.14)	40.60; 1; <0.001
Vaginal or anal penetration	224 (4.84)	64 (6.57)	160 (4.37)	8.07; 1; 0.004
Attempt of vaginal or anal penetration	153 (3.30)	50 (5.13)	103 (2.82)	12.94; 1; <0.001
Forcing to penetrate	54 (1.17)	24 (2.46)	30 (0.82)	18.04; 1; <0.001

Notes: Because the comparisons in this table involved 22 independent tests, we adopted a Bonferroni-corrected significance level of 0.05/22 = 0.002 for these analyses. The χ^2^ value refers to the comparison of SI minority vs. Non-SI-minority groups. Abbreviations: NR: Nationally Representative; SI: Self-Identified; SV: Sexual Violence.

**Table 5:** Occurrence of lifetime sexual victimization per minority group.

Lifetime SV	Sexual and Gender Minority ^1^ (*n* = 175) *n* (Valid%)	Cultural Minority ^2^ (*n* = 357) *n* (Valid%)	Other Minority ^3^ (*n* = 285) *n* (Valid%)	Multiple Minority ^4^ (*n* = 153) *n* (Valid%)	X^2^; df; *p*-Value
Any SV	137 (78.29) ^a^	237 (66.39) ^b^	186 (65.26) ^b^	121 (79.08) ^a^	17.05; 3; <0.001
Any hands-off SV	134 (76.57) ^a^	222 (62.18) ^b^	180 (63.16) ^b,c^	114 (74.51) ^a,c^	16.80; 3; <0.001
Any hands-on SV	72 (41.14) ^a,b^	111 (31.09)^b^	96 (33.68) ^b^	77 (50.33) ^a^	19.66; 3; <0.001
Sexual abuse	65 (37.14) ^a,b^	102 (28.57)^b^	87 (30.53) ^a,b^	65 (42.48) ^a^	11.55; 3; 0.009
Rape	36 (20.57) ^a,b^	36 (10.08)^c^	36 (12.63) ^b,c^	45 (29.41) ^a^	35.27; 3; <0.001

^1^ Individuals who selected at least one of the following characteristics for identifying in a minority group: Sexual orientation, gender identity and/or being an intersex/DSD person, and no characteristic from the other minority groups (cultural or other minority). ^2^ Individuals who selected at least one of the following characteristics for identifying in a minority group: Ethnicity, skin color, and/or religion/life philosophy, and no characteristic from the other minority groups (sexual and gender or other minority). ^3^ Individuals who selected at least one of the following characteristics for identifying in a minority group: Disability, age, and/or other characteristics, and no characteristic from the other minority groups (sexual and gender or cultural minority). ^4^ Individuals who are part of at least two of the above mentioned total groups for identifying in a minority group: Total Sexual and gender minority, total cultural minority and/or total other. A total of 34 respondents identified as being part of the sexual and gender minority as well as the cultural minority group, 68 respondents identified as being part of the cultural and other minority group, 32 as being part of the sexual and gender as well as the other minority group and 19 respondents identified themselves in all three groups. ^a,b,c^ The presented proportions per type of SV with different superscripts differ significantly (post-hoc χ^2^ test *p* > 0.05). Notes: Because the comparisons in this table involved five independent tests, we adopted a Bonferroni-corrected significance level of 0.05/5 = 0.010 for these analyses. Abbreviations: SV: Sexual Violence.

**Table 6:** Logistic regression analyses for two outcome variables: Lifetime prevalence of hands-off sexual violence and hands-on sexual violence.

	Hands-Off Sexual Violence				Hands-On Sexual Violence			
Predictors	Estimate	EXP(B) Odds Ratio	95% C.I. Odds Ratio	*p*	Estimate	EXP(B) Odds Ratio	95% C.I. Odds Ratio	*p*
**Sex assigned at birth** (ref. Female)				<0.001				<0.001
Male	−1.61	0.2	0.17–0.23		−1.02	0.36	0.31–0.41	
Age	−0.02	0.97	0.97–0.98	<0.001	−0.01	0.99	0.99–0.99	0.002
**Educational level** (ref. Primary education or none)				0.045				0.215
Secondary education	0.24	1.27	0.95–1.69		0.18	1.2	0.90–1.62	
Higher education	0.29	1.34	0.99–1.80		0.3	1.35	1.00–1.83	
**Occupational status** (ref. Remunerated workforce)				0.031				0.392
Student	−0.26	0.77	0.62–.96		−0.05	0.95	0.77–1.17	
Other	−0.19	0.83	0.69–0.99		−0.01	0.99	0.82–1.19	
**Financial situation** (ref. Perceived as easy)				<0.001				<0.001
Perceived as difficult	0.33	1.39	1.20–1.62		0.33	1.39	1.20–1.61	
**Sexual orientation** (ref. SI-heterosexual)				<0.001				<0.001
SI-LGB+ ^1^	0.52	1.68	1.24–2.28		0.62	1.85	1.42–2.42	
**SI-minority** (ref. No)				0.010				0.013
Sexual and gender minority ^2^	0.37	1.45	0.90–2.34		−0.08	0.92	0.61–1.38	
Cultural minority ^3^	0.16	1.17	0.92–1.51		0.05	1.05	0.82–1.35	
Other minority ^4^	0.39	1.47	1.12–1.94		0.24	1.28	0.97–1.67	
Multiple minority ^5^	0.49	1.64	1.07–2.54		0.57	1.77	1.21–2.58	

^1^ Participants who labeled themselves as: lesbian, gay, bisexual, omni-/pansexual, asexual, or other. ^2^ Individuals who selected at least one of the following characteristics for identifying in a minority group: Sexual orientation, gender identity, and/or being an intersex/DSD person, and no characteristic from the other minority groups (cultural or other minority). ^3^ Individuals who selected at least one of the following characteristics for identifying in a minority group: Ethnicity, skin color, and/or religion/life philosophy, and no characteristic from the other minority groups (sexual and gender or other minority). ^4^ Individuals who selected at least one of the following characteristics for identifying in a minority group: Disability, age, and/or other characteristics, and no characteristic from the other minority groups (sexual and gender or cultural minority). ^5^ Individuals who are part of at least two of the above-mentioned total groups for identifying in a minority group: Total sexual and gender minority, total cultural minority, and/or total other. A total of 34 respondents identified as being part of the sexual and gender minority as well as the cultural minority group, 68 respondents identified as being part of the cultural and other minority group, 32 as being part of the sexual and gender as well as the other minority group, and 19 respondents identified themselves in all three groups. Notes: Abbreviations: ref: reference category; SI: Self-Identified; LGB+: Lesbian, Gay, Bisexual, pan-/omnisexual, asexual, or other.

## Data Availability

The dataset supporting the conclusions of this article is available from the authors upon reasonable request.
